# Analysis of 1-Minute Potentially Available Fluoride from Dentifrice

**DOI:** 10.6028/jres.119.025

**Published:** 2014-12-04

**Authors:** Clifton M Carey, Erin C Holahan, Burton D Schmuck

**Affiliations:** University of Colorado Denver, School of Dental Medicine, Aurora, CO 80045; Bucknell University, Lewisburg, PA 17837; Washington State University, Pullman, WA 99164

**Keywords:** analysis, available fluoride, dentifrice, F-ISE, fluoride

## Abstract

Previous reports found that some fluoride-containing dentifrices do not release effective concentrations of fluoride during brushing. Failure to release fluoride can be due to dentifrice matrix components that interfere with the solubilization of the fluoride salts during brushing. A new generation of dentifrices has the capability to precipitate beneficial fluoride salts during tooth brushing. Therefore, a method that assesses the potentially available fluoride during the 1-minute brushing is needed. A new filter-paper absorption method to assess the 1-min bioavailable fluoride concentration was developed to meet this need. This method utilizes coiled filter paper that rapidly absorbs the aqueous phase of the dentifrice slurry followed by centrifugation to recover that fluid for fluoride measurement via fluoride ion-selective electrode. The analytical method was used to successfully determine the total fluoride and 1-min bioavailable fluoride in eight dentifrice products containing sodium fluoride (NaF), disodium monofluorophosphate (Na_2_FPO_3_, MFP), stannous fluoride (SnF_2_), or NaF with amorphous calcium phosphate (NaF + ACP). The results showed that some of the dentifrices tested had significantly lower potentially available fluoride than the total fluoride. For a MFP-containing sample, aged seven years past its expiry date, there was significant reduction in the bioavailable fluoride compared to MFP products that were not aged. Other than the aged MFP and the SnF_2_-containing samples the bioavailable fluoride for all products tested had at least 80 % of the label fluoride concentration. The filter paper absorption method yielded reproducible results for the products tested with MFP samples showing the largest variations.

## 1. Introduction

Fluoride-containing dentifrices have been in common usage since the 1960s when it was shown that dentifrices containing sodium fluoride (NaF), disodium monofluorophosphate (MFP), stannous fluoride (SnF_2_), acidulated phosphate fluoride, or amine fluoride reduced the caries rates in children [1]. It is generally agreed that the dentifrice must be able to release fluoride into the oral cavity during the time of tooth brushing to have anticaries efficacy [2, 3]. The American Dental Association in its acceptance program requires that 80 % of the labeled amount of fluoride must be released by the formulation with one minute of homogenization with a 1:3 dilution with water [4]. Recently some reports have found that there is a significant percentage of fluoride-containing dentifrice sold in the world market that does not release fluoride ions in sufficient amounts for caries prevention [5–7]. Fluoride salts in dentifrice can sometimes react with the individual dentifrice components including the abrasive, detergent, or other ingredients while in the original container to form insoluble fluoride salts that do not become available during use. Failure to release fluoride can be due to dentifrice matrix components that interfere with the solubilization of the fluoride salts during brushing [8]. These components regulate potential availability of fluoride, which can then affect clinical efficacy. When this happens, the fluoride is not available and thus the dentifrice may not have anticaries activity.

During tooth brushing, fluoride ions and/or compounds that hydrolyze to release fluoride ions (profluoride compounds) are released into the oral cavity. These are taken up by the oral tissues, precipitated into reservoirs as fluoride compounds, and later released as ionic fluoride. For this study, the definition of potentially available fluoride from dentifrice is: the amount of fluoride ion that becomes available in the oral cavity during and after tooth brushing with a fluoridated dentifrice. This includes the following forms of fluoride: ionic, precipitated, and profluoride compounds. MFP is an example of a profluoride compound that is hydrolyzed by alkaline phosphatase enzymes to release fluoride ion in the oral cavity [9, 10].

A number of methods describe the analysis of total fluoride in dentifrice [3, 11–13]. Analytical methods include titration, liquid chromatography, gas chromatography, ion chromatography, capillary electrophoresis and potentiometry (fluoride ion-selective electrode). The analysis of the fluoride that is available during tooth brushing requires that the method accounts for the need to solubilize the fluoride salt within the brushing time capturing the concentration of fluoride at that time as well as eliminating the possibility of fluoride reactions that could occur during the sample handling for analysis, for example, long centrifugation periods prior to analysis. Common methods for quantification of potentially available fluoride in dentifrice have been to suspend the dentifrice into a slurry for one minute, and then centrifuge the samples for ten minutes followed by analysis of the supernatants for fluoride content using the same techniques as for the total fluoride analyses [4]. These methods work well for the analysis of many NaF dentifrices where solubilized fluoride ions do not precipitate. However, analyses of MFP-containing dentifrices require an additional hydrolysis step prior to fluoride ion analysis. Over the recent decade a new generation of dentifrices has been introduced that incorporate chemistries resulting in the precipitation of fluoride reservoirs such as MFP or CaF-like deposits in dental plaque and oral soft tissues [14]. Many of these newer-generation dentifrices produce fluoride reservoirs within the first minutes of use. These fluoride reservoirs later release fluoride to the teeth over a longer period of time, which is thought to contribute to the products’ anticaries efficacy. Measurements of fluoride that do not account for these phenomena underestimate the potentially available fluoride. This is due to fluoride precipitation during the long centrifugation step resulting in lower fluoride concentrations in the supernate. Therefore a need exists to develop a robust method to quantify the potentially available fluoride from dentifrice at one minute.

Our studies have found that preparation of the dentifrice slurries and a rapid separation of the aqueous phase from the dentifrice slurry is essential for accurately determining the potential availability of fluoride from fluoride-containing dentifrices. Within the process, key steps require precise timing and measurement to allow for accurate results to be obtained. We present a method that allows for the rapid separation of an aqueous aliquot from the slurry. This method uses a coil of filter paper, which when introduced into the slurry at one minute, rapidly absorbs an aqueous aliquot. The aqueous phase is recovered from the filter paper via centrifugation through a filter, the filtrate is treated with acid to hydrolyze the sample, and analyzed for fluoride content. Precise preparation is important for allowing the samples to be measured with accuracy. Our hypothesis is that the rapid separation of free fluid from the dentifrice:water slurry will preclude the loss of potentially available fluoride due to precipitation of fluoride salts during sample preparation steps.

## 2. Materials and Methods

Dentifrices tested in this study were purchased in local supermarkets in Gaithersburg, MD.

### Materials

- TISAB II^1^ (Total Ionic Strength Adjustment Buffer) (Orion Research)
- Hydrochloric acid (HCl) 1 mol/L
- Potassium hydroxide (KOH) 1 mol/L
- Fluoride Ion-Selective Electrode (ISE) and meter
- TISAB Blank: Dilute TISAB II with deionized water 1:1
- NaF Standard solutions: (1 × 10^−2^, 1 × 10^−3^, 1 × 10^−4^, 1 × 10^−5^), mol/L NaF made in deionized water
- NaF Standard solutions: (1000, 750, 500) μg/g F (practical standards)
- Coiled rectangle of filter paper ~1.5 cm × 7 cm (e.g., Whatman Ashless #42)
- Centrifuge capable of spinning 1.5 mL microcentrifuge tubes at 12,000 g
- Centrifugal filter tube (Millipore: Microcon Ultrafree-MC 5.0 μm)
- Vortex mixer (e.g., Scientific Industries Vortex Genie)

### 1-Minute Sample Preparation

- Weigh centrifugal filter tubes without filter (M_T0_) then replace filter
- Combine dentifrice and distilled water: 1.0 g dentifrice with 3.0 g water
- Mix vigorously for 60 seconds by a combination of vigorous hand shaking and mixing with a vortex mixer. Note that the dentifrice must be fully suspended in the slurry for this assay; otherwise the measurement of available fluoride will be underestimated.
- Obtain 1-minute aqueous aliquot sample from the slurry:
  ▪ At 1 minute of mixing the slurry, submerge the coil of filter paper into the slurry for 15 seconds.
  ▪ Withdraw the wet filter paper and immediately insert into the centrifuge tube filter.
  ▪ Repeat the dipping of a second coil of filter paper (immediately after the first) to obtain a duplicate sample and insert it into a separate centrifuge tube filter.
  ▪ Immediately centrifuge the filters for 2 min at 12,000 g.
  ▪ The filtrate, collected in the bottom of the centrifuge tube, is the 1-minute potentially available fluoride sample.
  ▪ Save the slurry for the analysis of the total fluoride described below.
- Remove the filter, with the coil of filter paper, and weigh the centrifugal tube with filtrate (M_T1_).
- Calculate mass of filtrate (M_F_ = M_T1_ − M_T0_); assuming that the filtrate density is 1 g/mL, the volume of filtrate V_F_ [mL] is M_F_ [g].
- Add an equal volume (V_F_) of 1.0 mol/L HCl to the sample, mix and hydrolyze for at least 1 hour.
- Add an equal volume (V_F_) of 1.0 mol/L KOH to neutralize the hydrolyzed sample.
- Add 3 times the amount of sample (V_F_) of TISAB II to the sample, which is now ready for analysis.

### Sample Preparation for Total Fluoride Analysis

- Allow the slurry to stand for at least 5 minutes to allow for the liquid layer to separate from any foam.
- Remove 0.5 mL of the liquid layer and place in a test tube.
- Add 0.5 mL 1.0 mol/L HCl, mix and let stand for at least 1 hour.
- Add 0.5 mL 1.0 mol/L KOH and mix.
- Add 1.5 mL TISAB II and mix. This sample is ready for fluoride analysis.

### Fluoride Analysis via Fluoride Ion-Selective Electrode

- Calibrate a fluoride ion selective electrode (F-ISE) following the manufacturer instructions for the range of (1 × 10^−5^ to 1 × 10^−2^) mol/L NaF. The calibration plot (log[F^−^] mol/L vs potential (mV)) should be linear and have a slope that falls within the range of −56 mV to −60 mV per decade [F^−^] mol/L at room temperature.
- Place the F-ISE into 4.950 mL of TISAB blank solution (50 % TISAB/50 % H_2_O v/v) and when the mV potential indicates an F^−^ concentration less than 1 × 10^−5^ mol/L proceed to the next step.
- Add 50 μL of sample to the 4.950 mL TISAB blank and determine a stable mV potential within ± 0.1 mV for the diluted sample.

### Calculation of Fluoride Concentration

- Calculate the fluoride concentration [F^−^]_dil_ (mol/L) of the diluted sample from the standard curve made from the NaF standard solutions.
- Calculate the dentifrice fluoride concentration by multiplying [F^−^]_dil_ by the dilution factor of 1200 to obtain the [F^−^] (mol/L).
- Calculate the [F^−^] μg/g (ppm) by multiplying the calculated fluoride concentration, F^−^ (mol/L) by 19000 (μg·L)/(mol·g).
- Calculate the [NaF] μg/g (ppm) by multiplying the calculated fluoride concentration, F^−^ (mol/L) by 42000 (μg·L)/(mol·g).
- Note: Because the density of dentifrice is not 1.000 g/mL the calculation of [F^−^] μg/mL requires multiplication of the [F^−^] (μg/g) by the density of the product. Typical densities are given below for various dentifrice types:
  ▪ The density of NaF dentifrices is approximately 1.355 g/mL.
  ▪ The density of MFP dentifrices is approximately 1.500 g/mL.
  ▪ The density of SnF_2_ dentifrices is approximately 1.450 g/mL.
  ▪ The density of the dentifrice can be determined by filling a calibrated syringe with the dentifrice, dispensing a known volume of the dentifrice into a container (on a tared balance), and dividing the mass (g) by the volume (mL).

## 3. Standard Uncertainty Analyses

The greatest cause of variation in this analytical method comes from incomplete suspension of the dentifrice in the creation of the dentifrice-water slurry. We have found that if the dentifrice is not completely suspended in the slurry the resulting measured concentrations will be much lower than expected and have a high degree of variation. Another cause of variation comes from the dispensing of 50 μL of sample into the blank solution during fluoride measurement where the pipetted volumes can vary by several microliters resulting in 4 % variation in the apparent fluoride concentration. Finally, the fluoride ion-selective electrode analytical system will drift as much as a millivolt over the course of several hours. A 1 mV drift will cause a variation of F^−^ concentration of 40 μg/g for samples of F^−^ concentration of 1000 μg/g concentration. Other than electrode drift, each of these error sources can be minimized through careful sample handling during the preparation of the slurry, careful sample pipetting and including measurement of fluoride standards interspersed between sample measurements. Due to the F-ISE variations over time it is reasonable to assume that the standard uncertainty of this method is ± 3 % equal to the standard deviation of the F^−^ concentration of 1000 μg/g practical standard [15]. As a form of quality control, we suggest the inclusion of practical standards of F^−^ concentration of (500, 750, and 1000) μg/g that are treated in exactly the same manner as the dentifrice samples. The coefficient of variation as determined from 20 measurements of the practical F^−^ standard at 1000 μg/g concentration used in this analysis is 6.9 μg/g fluoride (0.7 %) which is less than the observed ± 3 % variation of the measurement technique.

## 4. Results

**Fig. 1:**
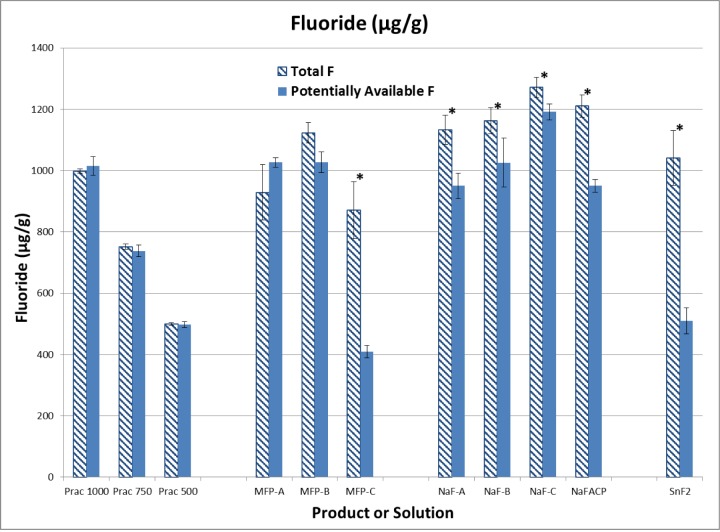
Total and potentially available fluoride comparisons for dentifrice and practical standards. The error bars shown are the standard deviations for each sample. ^*^Indicates a significant difference between total fluoride and potentially available fluoride concentrations (Student-t, p < 0.05).

Table 1 presents the total fluoride and 1-minute bioavailable fluoride concentrations measured in several different dentifrice products containing different sources of fluoride. The total fluoride measured was not significantly less than the product label for fluoride content for the dentifrices (Student-t at p ≤ 0.05) with the exception of an aged MFP-containing sample (sample MFP-C). The MFP-C product was from a tube of product that was 7 years past its expiration date. Several of the NaF and the NaF+ACP samples had significantly more total fluoride content than indicated on the product label, however these total concentrations were all less than the upper limit of 1500 μg/g fluoride. Figure 1 shows the comparison for total fluoride and potentially available fluoride for the samples. There was a significant difference between the total fluoride and potentially available fluoride measured for most of the samples indicating that a significant amount of fluoride was bound by the dentifrice ingredients. However, with the exception of the aged sample MFP-C, the potentially available fluoride for the MFP and NaF-containing products had at least 80 % of the label fluoride concentration and the SnF_2_-containing samples had almost 50 % of the label fluoride concentration, which all meet the current U.S. FDA requirements [16]. The variations between analyses were higher for the MFP samples where the hydrolysis of the solubilized MFP was necessary for the F-ISE measurements.

There was no statistical difference (Students-t, p ≥ 0.05) between the total fluoride concentration and the potentially available fluoride concentration measured for the practical standards. The recovery observed for these standards is (99.9, 100.3, and 100.0) % for the (1000, 750, and 500) μg/g fluoride practical standards, respectively.

## 5. Discussion

The results show that some of the dentifrices tested had significantly lower potentially available fluoride than total fluoride. Some of the samples analyzed in this study were several years out of date, representing challenging samples to analyze. The aged MFP sample (MFP-C) with calcium carbonate for the abrasive was 7 years past its expiry date and showed significant loss of potentially available fluoride. The method determined that one of the MFP and all the other samples (NaF, SnF_2_) had significant differences between the total fluoride and the potentially available fluoride measured; however the potentially available fluoride concentrations meet U.S. government requirements for fluoride availability [16]. The large loss of available fluoride due to dentifrice aging has been documented before [8] and this method validates this result.

The slurry ratio of 1 g dentifrice mixed with 3 mL water was chosen to mimic the dentifrice:saliva ratio used in the ADA guidelines for fluoride-containing dentifrice [4]. It has been suggested that the use of 0.1 mol/L K_2_HPO_4_ in place of water in the slurry will mimic the buffering capacity of saliva [17] and help reduce pH variations caused by the ingredients of the dentifrice products. Initial tests seem to support this suggestion.

Fluoride ion-selective electrodes respond to the fluoride concentration in a non-linear relationship following the Nernst Equation [18]. The Nernst Equation predicts a logarithmic relationship between the fluoride concentration and the measured potential. Therefore, the resolution of the F-ISE is best at low concentrations where small changes in fluoride concentration result in large changes in the measured potential. For example, if the sample is not diluted to a low concentration range then an error of ± 1.0 mV during the F-ISE measurement will result in a larger variation in the final calculated fluoride concentration than would occur if the analyte samples were more diluted. Each dentifrice should be run in triplicate (each sample is run in duplicate) to better estimate the product concentration. This is particularly important because a slight variation of ± 1 mV reading from the electrometer can result in a variation of as much as 40 μg/g fluoride for a 1000 μg/g fluoride dentifrice.

This method has not been tested with products that contain amine fluoride. Thus, we cannot recommend this method for the analysis of potentially available fluoride in dentifrice products that contain amine fluoride. Further, studies are needed to determine if there are additional steps needed for the method to be applicable for these types of dentifrices.

The use of coils of filter paper to absorb the free fluid from a dentifrice slurry is demonstrated to be a facile and rapid method to obtain a sample that is representative of the fluid in the oral cavity during brushing. This method of capturing the ionic fluoride and profluoride compounds prior to precipitation of fluoride salts accurately measures the potentially available fluoride concentration at 1 minute of slurry formation (i.e., tooth brushing). Although the volume of analyte recovered from the coil of filter paper is small, the concentration is such that the sample can be diluted by as much as 1:1000 rendering a volume that is easily analyzed by standard-size fluoride ion-selective electrodes. Additionally the sample recovered from the coils of filter paper could be analyzed by alternative methods which are available for fluoride analysis if needed. This method is designed for the analysis of potentially available fluoride in dentifrice samples that may have a kinetic limitation (delivers solubilized fluoride too slowly or the fluoride is quickly precipitated after release) and may not be necessary for the analysis of dentifrice that does not have kinetic limitations.

Some of the newer generation of dentifrices entering the market provide fluoride within the first minutes of use which then can precipitate into the oral tissues. These precipitates (fluoride reservoirs) later dissolve and release fluoride to the teeth over a longer period of time. Measurements of fluoride that do not account for these phenomena underestimate the potentially available fluoride. The intermediate steps of absorbing free fluid of the dentifrice slurry into a filter followed by quick removal via centrifugal filtration separates an aliquot of dentifrice slurry fluid before precipitation occurs and preserves the bioavailable fluoride for analysis. This modified method for determining bioavailable fluoride reduces the chances of underestimating potentially available fluoride from multifunctional dentifrice products. This modified method remains suitable for the analysis of products where precipitation of fluoride reservoirs does not occur.

## Figures and Tables

**Table 1:** Total and potentially available fluoride measured in dentifrice and practical standards

Dentifrice F source	Label F (μg/g)	Total F (S.D.) (μg/g)	Potentially Available F Mean (S.D.) (μg/g)	% Potentially Available F
Practical 1000 (n = 20)	1000	999 (7)	1015 (31)	101.7 (3.1)
Practical 750 (n = 11)	750	752 (8)	738 (20)	98.2 (2.7)
Practical 500 (n = 16)	500	500 (4)	498 (10)	99.6 (2.0)
MFP-A (n=6)	1000	929 (91)	1027 (16)	110.6 (9.8)
MFP-B (n = 4)	1000	1123 (47)	1028 (34)	94.4 (7.2)
MFP-C (n = 9)	1000	906 (101)‡	409 (20)*	47.0 (10.7)
NaF-A (n = 7)	1100	1133 (48)	950 (42)*	83.9 (4.3)
NaF-B (n = 7)	1100	1163 (42)‡	1026 (79)*	88.2 (7.7)
NaF-C (n = 6)	1100	1272 (33)‡	1192 (26)*	93.7 (7.6)
NaF-ACP (n = 6)	1100	1211 (36)‡	950 (21)*	78.5 (3.0)
SnF_2_ (n = 7)	1100	1041 (90)	510 (42)*	48.9 (8.6)

S.D. is the standard deviation.

‡ indicates a significant difference between the total fluoride measured and the total fluoride indicated on the product label (Student-t, p ≤ 0.05).

* indicates a significant difference between the total fluoride and potentially available fluoride concentrations (Student-t, p ≤ 0.05).

## Footnotes

1 Certain commercial materials, equipment, and software are identified in this paper in order to specify adequately the experimental and analysis procedures. In no case does such identification imply recommendation or endorsement by the National Institute of Standards and Technology (NIST) the American Dental Association Foundation, or the University of Colorado Denver nor does it imply that they are necessarily the best available for the purpose.
